# Pyorrhœa Alveolaris

**Published:** 1886-09

**Authors:** Alfred R. Starr


ARTICLE II.

### PYORRHŒA ALVEOLARIS.

BY ALFRED R. STARR, M. D., D. D. S.

Pyorrhœa Alveolaris, sometimes called Rigg's disease, catarrhal or suppurative gingivitus or ulitis, and alveolar pyorrhœa, is a disease of which much has been written, but as yet little is known. It is described by some as a disease characterized by a flow of pus from the tooth sockets. The effect upon the gums and alveoli differs very materially from the usual results of salivary calculus, in that in this disease the ulcerative process or retrograde metamorphosis is most marked in the pericementum and alveolus, while the gums are comparatively free. In this affection the destruction and separation of the pericementum and the absorption of the alveoli are greater and more rapid than the recession of the gums, thus resulting in the formation of deep pockets between the gums and the teeth, from which pockets exudes an ichorous or sanious discharge, In cases of salivary calculus proper, with no secondary sanguinary deposit, the recession of the gums, destruction of the pericementum and absorption of the alveolus occur slowly, and the process is limited to the immediate vicinity of the deposit; so that if we go a little beyond the point of contact of the deposit with the gum, we will find the pericementum and alveolus in quite a normal condition.

This is the case, even when the deposit has encroached upon the alveoli almost to the apices of the roots. Even in these cases we may have a fœtid, sanious discharge, but instead of proceeding from deep pockets it comes from the tissues in the immediate vicinity of and directly underlying the deposit. If any pockets are formed in these cases of salivary calculus they are very shallow, and the destruction of the pericementum and absorption of the alveoli show little tendency to increase any more rapidly than the ulceration and recession of the gums. In pyorrhœa alveolaris there is frequently no recession of the gums, little or no salivary calculus about the necks of the teeth, and yet we have extensive involvment of the pericementum and alveolus and usually, if not always, the presence of the dark or sanguinary variety of tartar on the roots.

We sometimes see the manifestations of these two affections, viz: salivary calculus and pyorrhœa alveolaris, on one and the same tooth. In the case of salivary calculus proper, the deposit precedes and causes destruction of the pericementum, while in this disease some peculiar irritation of the pericementum precedes and causes calcareous deposit.

The etiology of pyorrhœa alveolaris is very obscure. Authorities are very evenly divided as to whether the causes are constitutional or local. Some regard it as a localization of a systemic debility, while others believe it to be due entirely to local causes, and amenable to local surgical treatment. Some attribute the occurrence of the disease entirely to the presence of tartar and its effects upon the surroundings of the teeth, while others say that while tartar is usually present it is only a concomitant or sequence of the affection and never the cause. Those who maintain the latter view declare that the disease sometimes occurs without the presence of any tartar.

Constitutional dyscrasia (hereditary or acquired), extreme density and low degree of vitality of the teeth, suppression of habitual secretions, catarrhal inflammation, the presence of bacteria, of foreign deposits (salivary, serumal or sanguineous), and local irritation from the use of wedges, ligatures, rubber dam, &c., have been assigned as causes.

The influence of heredity in pyorrhœa is often quite marked, the disease being transmitted through several generations. Cases have been noticed in which children born before the acquisition of the disease by the parent or parents have been exempt, while those born subsequently have developed it at quite an early age. Among the cases due to acquired constitutional predisposition may be cited those caused by mercurialization, or some peculiarity of diet, nutrition, or nervous influence.

Pyorrhœa alveolaris very frequently follows mercurial salivation. The statement has been made that pyorrhœa alveolaris never occurs except in persons who have been salivated, but this theory has not been generally accepted, and I do not believe it is founded on fact.

It is believed by many that excessive use of chloride of sodium will sometimes cause pyorrhœa alveolaris. Some authors assert that imperfect elimination of urea is its principal constitutional antecedent.

In support of the theory that suppression of habitual secretions may aggravate or incite this affection, Dr. Rehwinkel cites the case of a young lady aged eighteen, otherwise healthy, and with no accumulation of salivary calculus, in whom the teeth became very loose, presumably from the fact that the menses had never been established. The extraction of two or three of her teeth, although they were very loose, produced violent and persistent hæmorrhage. Local treatment and hygienic measures checked the progress of the malady, and when, after some months, menstruation was established, the disease disappeared and the remaining teeth became firm. Dr. Patterson has said that he believes the disease to be of a catarrhal nature, and he also inclines to the belief that the calcular deposits are simply the result or sequence of the disease. Dr. Patterson states that in the cases he has observed he has found co-existing nasal, pharyngeal, or laryngeal catarrh (generally combined) in every instance. He believes the disease is generally caused by infection from a pre-existing catarrh of the nose or throat, but states, also, that the catarrhal condition of the mouth may originate in that cavity, and not be due to infection, or (I think he should have said) extension of the disease, at all. These primary cases, he thinks, are most apt to occur in those who are in the habit of breathing through the mouth. In support of his theory Dr. Patterson cites the following points of similarity in the pathology of the two diseases, viz:—Nasal catarrh and pyorrhœa alveolaris.

1st. The similar appearance of the affected mucous membrane in both diseases and in the various stages of each.

2nd. The identical character of the effusions, viz: first serous, containing numerous epithelial scales, and then becoming filled with pus and blood corpuscles.

3rd. The infectious nature of both diseases, nasal catarrh being contagious and sometimes epidemic, pyorrhœa alveolaris frequently showing a tendency to spread from one tooth to the next, until all may be affected.

4th. The similar burrowing of pus in each trouble.

5th. The tendency in each to destruction of periosteum and underlying bone.

6th. The calcareous deposits occurring in each disease. (Deposits of phosphate and carbonate of lime are sometimes formed in cases of nasal catarrh.)

It is possible that the predisposing or constitutional cause of pyorrhœa alveolaris may, in some instances, be a tendency to catarrhal inflammations; but I do not believe, as does Dr. Patterson, that this disease is transmitted from a pre-existing catarrh of the nose or throat. It is true we can have, according to the medical authorities, an extension of catarrhal inflammation from the nose, throat, or even from the stomach, to the mouth, and we then have acute or chronic oral catarrh, or catarrhal stomatitis; but in such cases the process is a general one, and affects not only the mucous membrane of the gums, but also that of the lips, cheeks, tongue, &c., which condition we do not have in pyorrhœa alveolaris. Dr. Patterson states that both nasal catarrh and pyorrhœa alveolaris are of an infectious nature, and further states that text-books all agree that nasal catarrh is not only contagious, but sometimes epidemic.

There may be some instances in which the disease appears to be infectious. The epidemic said to have occurred in St. Gall, Switzerland, in 1876, if the reports be authentic, would be an instance of this kind. In this epidemic the disease was said to be very severe, and investigation demonstrated the presence of numerous parasites (leptothrix, bacteria, &c.) in the secreted matter, but no pus corpuscles. Schlenker, who studied these cases, concluded that the presence of the parasites was the cause of the inflammation of the root membrane. Some observers, among them being Dr. G. V. Black, of Illinois, and Dr. Witzel, of Germany, believe that the disease is caused by a certain species of fungus. We cannot deny the possibility of such a mode of origin, although I think no one has yet been able to demonstrate, by actual experiment, that there is any specific virus or contagium in this disease. I have endeavored to transmit the disease by inoculation from the human subject to the dog, but so far have been unable to produce anything except a negative result. My method has been to make a slight incision between the gum and the prominent cuspid tooth of the dog, and inoculate with the fresh discharge carried on an instrument directly from the patient to the animal in the next room. It may be that the lower animals are not capable of developing the disease. It might be interesting and instructive to experiment in the mouth of a patient affected with the disease, by inoculating from the socket of an affected tooth to one not affected (by applying the discharge to a denuded surface), and observing the result. I have not as yet experimented in this manner to any great extent. Dr. Patterson states that there is the same tendency to destruction of periosteum and underlying bone in this disease as in nasal catarrh. I beg to differ with him in regard to the tendency to destruction of bone in pyorrhœa alveolaris. In nasal catarrh, when the bone is involved the process is one of caries, or necrosis, while in pyorrhœa alveolaris I think it is only very rarely that we have such a condition; but of this we shall speak further in treating of the pathology of the disease.

Whether or not there is always a constitutional predisposition in cases of this disease, is still a matter of much controversy. The preponderance of opinion seems to be that there is usually a constitutional predisposition. The traumatic or acute cases, without doubt, are due entirely to local causes, since almost any irritant of the kind described will induce the disease, and the removal of the cause results in a speedy cure. Cases in which salivary calculus irritates the pericementum and causes secondary sanguinary deposit might be classed as traumatic, for the exciting cause is a foreign body; but, although to a certain extent traumatic, they cannot be called acute cases, since the disease when induced by this cause generally assumes the chronic form. Perhaps this may be accounted for by the fact that the salivary deposit increases very gradually, and the irritation is less on that account. I think that even in these cases of pyorrhœa from the irritation of salivary calculus, we must admit the presence of a constitutional predisposition (I refer now to typical cases, in which we have the deposit of sanguinary calculus and the formation of ulcerating and suppurating pockets); because we know that not all cases of salivary calculus, or of pericemental irritation, are followed by this disease. In fact, it results in comparatively few instances.

It is difficult to draw the line between local and constitutional origin in these cases, for if the exciting cause be salivary calculus, that in itself is often dependent upon constitutional derangement for its development. The cases in which we find no appreciable exciting cause are the ones less amenable to treatment.

To sum up, then, we regard the traumatic or acute cases as essentially local in their origin, since they are so easily induced and are so readily amenable to local treatment without showing any tendency to recurrence; but in the idiopathic or chronic cases, or those of pyorrhœa alveolaris proper, we think the causes are both predisposing and exciting, and that there is generally, at least, a constitutional predisposition rendering the disease liable to occur under local irritation, either mechanical or chemical. Salivary calculus, I think, is the most common exciting cause. The irritation of partial plates would probably come next in order of frequency. What the predisposing cause or causes may be, we are as yet not aware. Possibly the same, or a similar influence to that which causes exostosis of the cementum, may operate in determining the origin of this affection, the difference being, that in this disease the lime salts, instead of helping to form an organized tissue, are deposited in an amorphous manner. I think I have met with a greater number of cases affecting the teeth of the superior maxilla than of the inferior; but whether or not this has been the experience of other observers, I am not aware. The disease is one of adult life, and is common to both sexes. It is very rare in young persons, except when hereditary.—*Independent Practitioner.*

